# Supplementary material for: Deconstruction of the beaten Path-Sidestep interaction network provides insights into neuromuscular system development
Source: eLife. 2017 Aug 15;6:e28111. doi: 10.7554/eLife.28111 (PMC5578738; doi:10.7554/eLife.28111)
Supplement: Transparent reporting form [file elife-28111-transrepform.pdf]

eLife's transparent reporting form

We encourage authors to provide detailed information *within their submission* to facilitate the interpretation and replication of experiments. If you have any questions, please contact us: editorial@elifesciences.org.

Sample-size estimation

- You should state whether an appropriate sample size was computed when the study was being designed
- You should state the statistical method of sample size computation and any required assumptions
- If no explicit power analysis was used, you should describe how you decided what sample (replicate) size (number) to use

Please outline where this information can be found within the submission (e.g., page numbers or figure legends), or explain why this information doesn't apply to your submission:

This issue comes into play at only one point in the paper, in which we analyzed the side-VI phenotype. Based on our extensive prior experience with motor axon guidance, we used a sample size of >200 hemisegments, which we knew would be sufficient to attain statistical significance even for a low-penetrance phenotype. On p. 18, we report that the p-value for the difference between side-VI and controls is 2.53 x 10e7.

Replicates

- You should report how often each experiment was performed
- You should include a definition of biological versus technical replication
- The data obtained should be provided and sufficient information should be provided to indicate the number of independent biological and/or technical replicates
- If you encountered any outliers, you should describe how these were handled
- Criteria for exclusion/inclusion of data should be clearly stated
- High-throughput sequence data should be uploaded before submission, with a private link for reviewers provided (these are available from both GEO and ArrayExpress)

Please outline where this information can be found within the submission (e.g., page numbers or figure legends), or explain why this information doesn't apply to your submission:

eLife Sciences Publications, Ltd is a limited liability non-profit non-stock corporation incorporated in the State of Delaware, USA, with company number 5030732, and is registered in the UK with company number FC030576 and branch number BR015634 at the address 1st Floor, 24 Hills Road, Cambridge CB2 1JP | August 2014

On p. 26, we state that in the Bio-Plex analysis, each binding reaction was run in duplicate. This is necessarily a technical replication, as biology is not involved here; this is an in vitro binding experiment. We did not exclude any data. All raw signals are shown in Figs. 2B and 2C. In Results and Materials and Methods, we explain how the raw data were processed to generate the conclusions shown in Fig. 3.

Statistical reporting

- Statistical analysis methods should be described and justified
- Raw data should be presented in figures whenever informative to do so (typically when N per group is less than 10)
- For each experiment, you should identify the statistical tests used, exact values of N, definitions of center, methods of multiple test correction, and dispersion and precision measures (e.g., mean, median, SD, SEM, confidence intervals; and, for the major substantive results, a measure of effect size (e.g., Pearson's r, Cohen's d)
- Report exact p-values wherever possible alongside the summary statistics and 95% confidence intervals. These should be reported for all key questions and not only when the p-value is less than 0.05.

Please outline where this information can be found within the submission (e.g., page numbers or figure legends), or explain why this information doesn't apply to your submission:

For the in vitro binding data, the procedure for analysis of raw data and extraction of meaningful binding interactions is explained in detail on pp. 12=13 and pp. 27-28.
For the motor axon guidance phenotypes, we have reported the p-value on p. 18. No-one in the field reports 95% confidence intervals or describes statistical tests in detail for this kind of phenotypic analysis, as the statistics are simple and self-evident.

(For large datasets, or papers with a very large number of statistical tests, you may upload a single table file with tests, Ns, etc., with reference to page numbers in the manuscript.)

Additional data files ("source data")

- We encourage you to upload relevant additional data files, such as numerical data that are represented as a graph in a figure, or as a summary table
- Where provided, these should be in the most useful format, and they can be uploaded as "Source data" files linked to a main figure or table
- Include model definition files including the full list of parameters used
- Include code used for data analysis (e.g., R, MatLab)
- Avoid stating that data files are "available upon request"

Please indicate the figures or tables for which source data files have been provided:
